# The Bioaccumulation and Biodegradation of Testosterone by *Chlorella vulgaris*

**DOI:** 10.3390/ijerph16071253

**Published:** 2019-04-08

**Authors:** Mei Fu, Bixiang Deng, Hongjian Lü, Weizhi Yao, Shengqi Su, Dingyong Wang

**Affiliations:** 1 Key Laboratory of Freshwater Fish Reproduction and Development (Ministry of Education), College of Animal Science and Technology, Southwest University, Chongqing 400715, China; fumei@swu.edu.cn (M.F.); ymgdymail@163.com (B.D.); hongjianlv@swu.edu.cn (H.L.); yaowz@swu.edu.cn (W.Y.); 2 College of Resources and Environment, Southwest University, Chongqing 400715, China; dywang@swu.edu.cn

**Keywords:** bioaccumulation, biodegradation, testosterone, environmental androgens, *Chlorella vulgaris*

## Abstract

In the present study, the accumulation and degradation of testosterone by *Chlorella vulgaris* were studied. The results showed that *C. vulgaris* has a significant ability to eliminate testosterone by bioaccumulation and biodegradation, and during the 96 h experimental period, the data demonstrated that the accumulation of testosterone followed a sigmoidal accumulation pattern. At the end of the experiment, the bioconcentration percentages of testosterone by *C. vulgaris* in the high-concentration group and the low-concentration group were 11.49 ± 2.78% and 40.10 ± 1.98%, respectively, and the biodegradation percentages of testosterone were 69.64 ± 4.33% and 42.48 ± 1.92%, respectively. The rate of biodegradation of testosterone by *C. vulgaris* mainly depended on the relative initial concentration of testosterone. When the relative initial concentration of testosterone increases, the degradation may gradually change from zero-order kinetics to second-order kinetics.

## 1. Introduction

Environmental androgens are a class of typical endocrine disruptors [1,2] that can interfere with the normal endocrine system of organisms even at trace levels [3,4]. Moreover, environmental androgens are detrimental to the reproductive development of aquatic organisms, affecting the structure and function of microbial communities [5,6,7,8]. Environmental androgens have a wide range of sources [9,10,11,12,13,14] and can migrate to subterranean and surrounding environments through infiltration and runoff [15,16], thereby increasing the risk and scope of contamination.

Testosterone, as a representative environmental androgen, is mainly derived from human and vertebrate emissions [13,17]. In recent years, due to the continuous discharge of pollution sources, testosterone has been detected and reported in various environmental media (surface water, groundwater, river, sediment, etc.) in many countries and regions [13,17,18]. The ecological risk can therefore not be underestimated. Studies have shown that testosterone can interfere with the normal endocrine system of aquatic organisms, causing a high proportion of males in some aquatic organisms [19], the appearance of male secondary sexual characteristics in female fish, the inhibition of vitellogenin induction, and the reduction of reproductive capacity [5,20]. Testosterone can also cause masculinization to arise in mammals [21,22]. Therefore, the migration and accumulation of testosterone in environmental media poses a serious threat to the health of the ecological environment. Research by Jacobsen et al. [23,24,25] showed that biodegradation is the major process for the removal of environmental androgen. Therefore, research on the biodegradation of testosterone is of profound significance for pollutant treatment and ecological risk assessment.

As a primary producer of aquatic ecosystems, single-cell microalgae can quickly accumulate pollutants from the water. In addition, as a point of entry of pollutants within trophic nets, the accumulation and degradation of pollutants by single-cell microalgae will have an impact on organisms of higher trophic levels. Therefore, research on the bioaccumulation of environmental androgens in microalgae can provide a scientific basis for the study of the ecological effects of environmental androgens.

Many studies have reported the removal and biodegradation of organic contaminants by microalgae. For example, *Raphidocelis subcapitata* has been used to remove 17β-estradiol and diethylstilbestrol [26], and *Scenedesmus obliquus* has been shown to have the ability to remove and degrade climbazole [27]. Additionally, *S. quadricauda*, *Chlorella vulgaris*, *Ankistrodesmus acicularis*, and *Chroococcus minutus* have a reported rapid and high ability to remove nonylphenol [28]. However, few studies on the migration and degradation of environmental androgens in aqueous environments have been reported.

*C. vulgaris* is a single-cell green microalga commonly found in freshwater environments that is easy to grow in the laboratory and is widely used in organic pollutant treatment research, which has provided excellent results in recent years [29]. For example, *C. vulgaris* showed potential removal capability for potassium cyanide, with a maximal removal rate of 61% [30]. The biotransformation and bioconcentration of natural and synthetic steroid estrogens by *C. vulgaris* has also been reported [31]. Therefore, *C. vulgaris* has the potential ability to remove testosterone. The present study aimed to investigate the testosterone bioaccumulation and biodegradation capacity of *C. vulgaris* under monoalgal culture conditions and to briefly explore the degradation kinetics.

## 2. Materials and Methods

### 2.1. Algae and Culture Condition

*Chlorella vulgaris* was obtained from the Institute of Hydrobiology, Chinese Academy of Sciences, Wuhan, Hubei, China. The unialgal stock was maintained in 250 mL flasks containing 150 mL of BG11 medium [32] at 25 ± 1 °C under a 12 h:12 h light:dark cycle with 60 μmol photon m^−2^s^−1^ during the light period in an illumination incubator. The glassware was washed to remove possible contaminants, thoroughly rinsed with distilled water, and then autoclaved at 121 kPa for 30 min. 

### 2.2. Experimental Design

The testosterone (Sigma-Aldrich, purity >98%, St.Louis, MO, USA) toxicity experiment on *Chlorella vulgaris* was conducted previously. Experimental data showed that half of the maximum effective concentration (EC50) of testosterone that induced the inhibition of growth of *C. vulgaris* was approximately 58 mg/L after 96 hours and that at a concentration of 3 mg/L (α = 0.05) the testosterone did not show significant inhibiting effects. Therefore, also considering the environmental concentrations of testosterone, two treatment groups were created: a high-concentration group and a low-concentration group, with initial testosterone concentrations of 0.2 mg/L and 0.02 mg/L respectively. The initial density of the algal cells was 5 × 10^6^ cell/mL. The cells were cultured under identical conditions as the stock culture. Three control groups were established simultaneously, Control I and Control II without algal cells to rule out the natural degradation of testosterone, and Control III receiving only algal cells to be a control for algae growth and to rule out the testosterone from the surrounding air and the algae and liquid culture media for inoculation. The other conditions were the same as those of the experimental groups (Table 1).

At specific time points (0 h, 0.5 h, 1 h, 3 h, 6 h, 12 h, 24 h, 48 h, and 96 h), samples were collected to analyze the density of algal cells and testosterone concentrations both in the water and in the cells. The reported values are the average observed values of three parallel samples. 

### 2.3. Algal Density

A spectrophotometer (METASH, UV6100A, ShangHai, China) was used to scan the characteristic absorption wavelengths of the algal cell suspensions and measure the absorbance in different algal suspension densities (wavelength = 680 nm) [33]. At the same time, a hemocytometer was used to count the number of algal cells per unit volume under a microscope. Then, a standard curve was established between the algal cell number per unit volume and the absorbance (*R*^2^ = 0.999). 

### 2.4. Dry Weight of Algal Cell

A 10 mL algal suspension with known density was centrifuged in tubes that had been pre-dried to a constant weight at 103 °C. Then, the centrifuge tubes were dried to a constant weight again, cooled in a vacuum desiccator, and weighed to obtain the total dry weight. The cell number of the algae pellet at the bottom of the centrifuge tubes was calculated by the difference of the observed number of the algal cells in the algal suspension before and after centrifuging. The dry weight of a single algal cell was determined by dividing the total dry weight by the number of algal cells.

### 2.5. Determination of Testosterone in Algal Cells

The centrifuge tube that contained 5 mL algal suspension was centrifuged at 4000 rpm for 5 min to eliminate the supernatant. The algae pellet at the bottom of the centrifuge tube was extracted using 3 mL of n-hexane (repeated three times) after abrading by a pestle at 1000 rpm for 10 min. The testosterone-n-hexane solution was filtered through a 0.22 µm membrane and analyzed via HPLC-MS. The average extraction recovery was 86.23 ± 1.33%. The dry weight of an algae pellet at the bottom of the centrifuge tube was calculated by the dry weight of a single algal cell multiplying by the algal cell number of the algae pellet. Then the concentration of testosterone in algae (dry weight) could be calculated.

### 2.6. Determination of Testosterone in Water

The filtrate from 5 mL of the algal suspension filtered with a 0.45 μm membrane was extracted by sonicating with 3 mL of n-hexane for 3 min for a total of three repetitions. Then, the testosterone-n-hexane solution was filtered through a 0.22 µm membrane and was determined via HPLC-MS. The average extraction recovery of the analytical methods was 92.59 ± 1.23%, which was subsequently used to correct the observed testosterone concentrations in the experiments. 

The concentration of testosterone in the algae–water system is the sum of testosterone measured in the water and algal cell fractions.

### 2.7. HPLC-MS Conditions

Testosterone levels were quantified using a Waters UPLC-Q-TOF-MS ACQUITYUPLC system (Waters Corp., Milford, MA, USA) in positive electrospray (ESI) and sensitivity mode. An ACQUITY BEH C_18_ column (100 mm in length with an inner diameter of 2.1 mm, 1.7 μm particle size, Waters Corp, Milford, MA, USA) was used at a constant flow rate of 0.4 mL/min. The mobile phase consisted of 0.1% formic acid in Milli-Q water (A) and acetonitrile (B). The injection volume was 1.5 μL. The capillary voltage and cone voltage were 3000 V and 40 V, respectively, while the desolvation temperature and source temperature were 400 °C and 120 °C, respectively. The gas flows of the cone and desolvation were 50 L/h and 800 L/h, respectively. The collision energy ramp for the high energy function ranged from 20 to 40 eV. All the sample data were processed using MassLynxTM4.1 software (Waters Corp., Milford, MA, USA). 

### 2.8. Statistical Analysis

Statistical analysis was performed using SPSS 23.0 statistical software (SPSS Inc., Chicago, IL, USA). The experimental data were analyzed using one-way analysis of variance (ANOVA), followed by Dunnett’s test to compare the significance of the treatments. Significance testing was performed on all data and the significance level used for all statistical tests was 0.05. The data are shown as the means ± standard errors (SE).

## 3. Results

### 3.1. Algal Cell Growth

The algae cells were sampled and tested nine times. The algal density of *Chlorella vulgaris* under different concentrations of testosterone is presented in Table 2. No statistically significant differences were observed among the treatment groups and the Control III group with regard to algal cell growth during the experimental period (*p* > 0.05). Thus, the test concentrations of testosterone in the following experiment of accumulation and degradation were reasonable.

### 3.2. Accumulation of Testosterone in Chlorella Vulgaris

**Figure 1:**
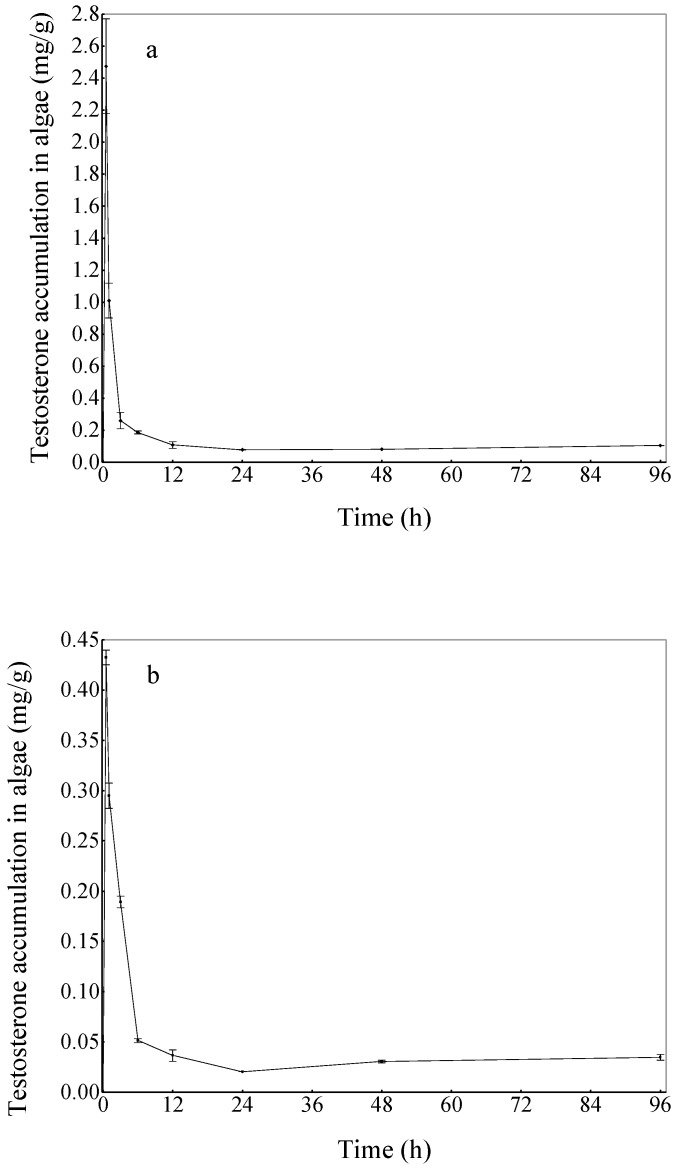
(**a**) The processes of testosterone accumulation by *Chlorella vulgaris* in the high-concentration group. (**b**) The processes of testosterone accumulation by *C. vulgaris* in the low-concentration group. The vertical bars represent the standard error. For the data points without an error bar, the error bar is smaller than the symbol.

The dynamic variation in the amount of accumulated testosterone in algal cells over time is presented in Figure 1. In the treatment groups, both the high-concentration group and the low-concentration group demonstrated a sigmoidal accumulation pattern for testosterone (Figure 1a, Figure 1b). During the 96 h experimental period, there was a significant and rapid accumulation during the initial phase, and the concentration of testosterone in the algae (dry weight) reached a maximum at the 0.5 h sampling points for both the high-concentration group and low-concentration group (Figure 1). Then, the concentration of testosterone in the algae began to decline at the end of the initial rapid sorption process, after which a slower accumulation process began to dominate. At the last sampling point of the experiment, compared to the initial concentration, the testosterone amounts of the high concentration group and the low concentration group detected in algal cells were 11.49 ± 2.78% and 40.10 ± 1.98%, respectively. Testosterone was not detected in the Control III group.

### 3.3. Degradation of Testosterone by Chlorella Vulgaris

**Figure 2:**
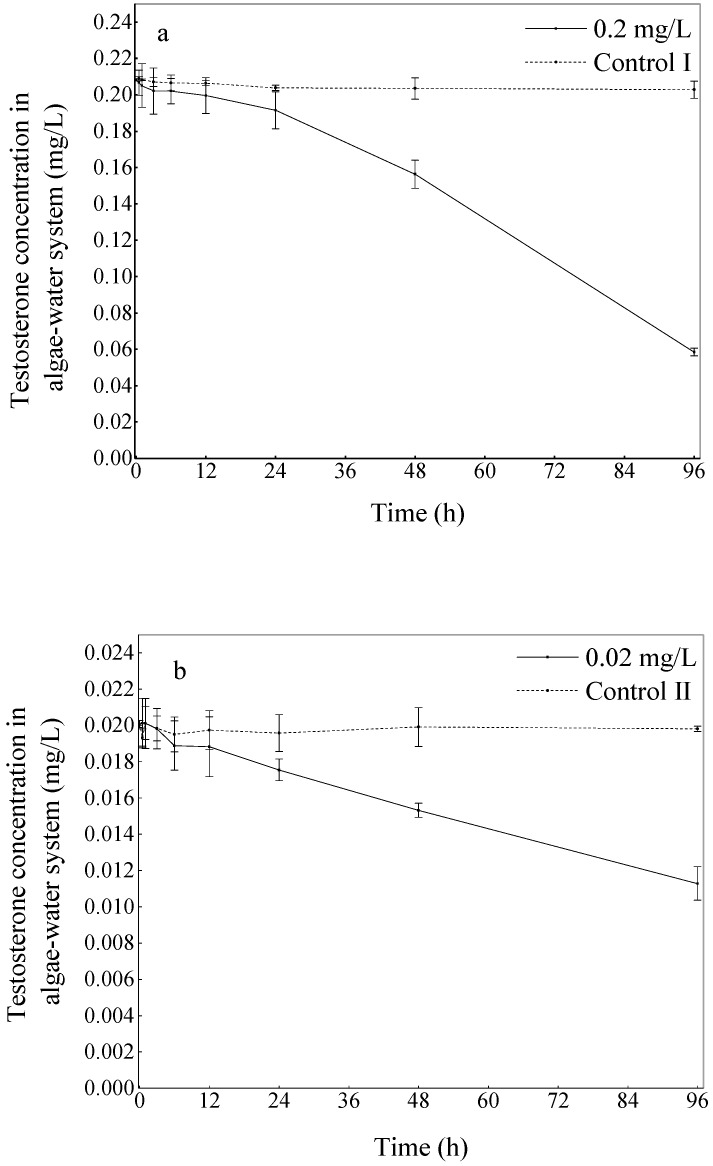
(**a**) The processes of testosterone biodegradation by *Chlorella vulgaris* in the high-concentration group. (**b**) The processes of testosterone biodegradation by *C. vulgaris* in the low-concentration group. The vertical bars represent the standard error. For the data points without an error bar, the error bar is smaller than the symbol.

The variation in the total testosterone concentrations in the algae–water systems are shown in Figure 2. During the 96 h test period, the testosterone concentrations in the high-concentration group and low-concentration group were reduced by 69.64 ± 4.33% and 42.48 ± 1.92%, respectively, compared with the initial concentration.

### 3.4. The Kinetics Equation of Degradation

Yan et al. [34] suggested that the biodegradation rate of hydrophobic organic chemicals (HOCs) is affected by the algal cell density and growth rate at different growth stages based on the tests on the degradation of three lipid compounds by *Chlorella pyrenoidosa*. For the results of this experiment, a second-order reaction kinetics equation was used to determine the testosterone biodegradation characteristics. The kinetics equation for the testosterone degradation speed was expressed as follows:

(1) $$\frac{d\left[Tes\right]}{dt}=KNr$$

where [*Tes*] is the testosterone concentration, *K* is a constant, *N* is algal cell density, and *r* is the rate of algal growth. 

An enhancement was made to Equation (1) in which *r* was expressed as *dN*/*dt*. After solving Equation (1), then:

(2) $$\frac{d\left[Tes\right]}{dt}=KN\frac{dN}{dt}$$

(3) ∫d[Tes]=K∫NdN

(4) $$\left[Tes\right]=\frac{1}{2}KN^{2}+C$$

where *C* is a constant. 

The constant *K* and the constant *C* can be obtained by a linear regression of Equation (4) between the observed concentrations of testosterone and the square of the observed densities of *C. vulgaris* cells at different periods. Changes in the testosterone concentration in the algae–water system with time can be imitated by Equation (4) after substituting the obtained *K* and *C* values. 

**Figure 3:**
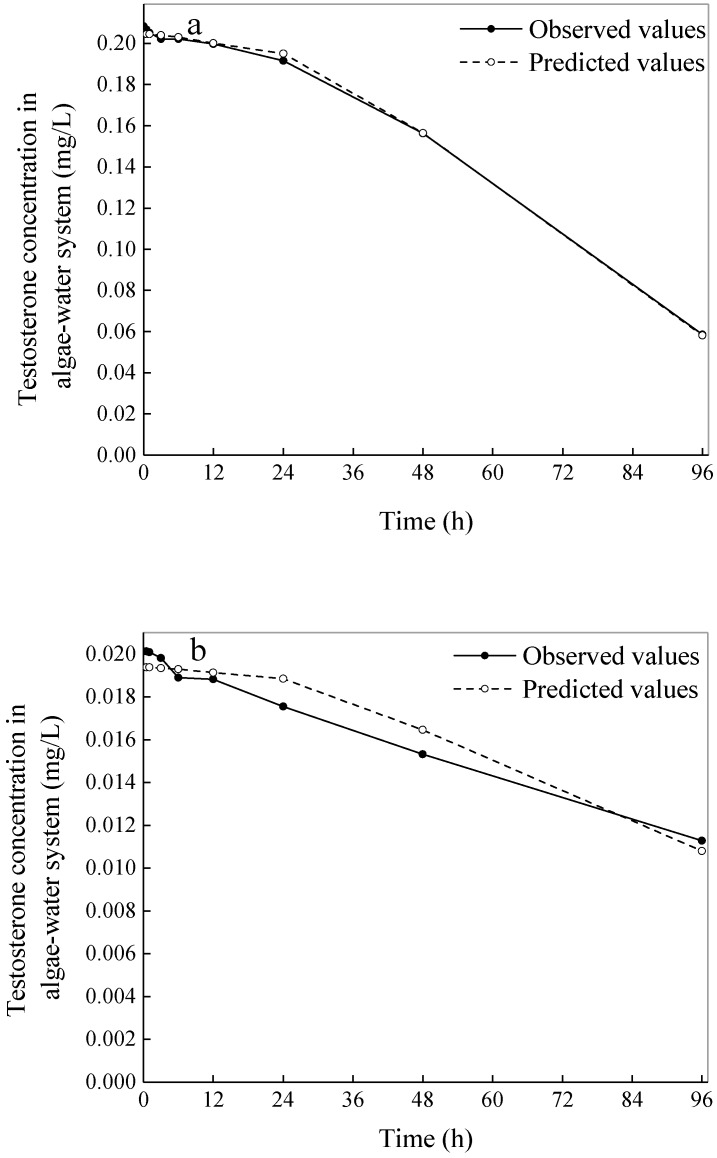
(**a**) The processes of testosterone biodegradation by *Chlorella vulgaris* with observed values and predicted values fit by Equation (4) in the high-concentration group. (**b**) The processes of testosterone biodegradation by *C. vulgaris* with observed values and predicted values fit by Equation (4) in the low-concentration group.

The relationships between [*Tes*] and *t*, both the observed values and predicted values, are shown in Figure 3. The results calculated by Equation (4) indicate that the relative mean deviation of testosterone concentration in the algae–water system of the 0.2 mg/L group between the calculated values and the observed values was 0.79%, *r* = −0.999. This result suggests that this kinetic equation could well describe the process of testosterone biodegradation by *C. vulgaris* with the experimental data of the 0.2 mg/L group. In the 0.02 mg/L group, the relative mean deviation and *r* value were 4.14% and −0.962, respectively (Figure 3). This poor accuracy and objectivity were obviously due to the relatively low testosterone concentrations in the lab samples. Under this circumstance, the rate of testosterone biodegradation is presumed to be minimally affected by the density of the algae cells. Even when the testosterone concentration is very low, the growth rate of the algal cells could also be negligible. Under that assumption, differential Equation (1) can be reduced to:

(5) $$\frac{d\left[Tes\right]}{dt}=Kr$$

or

(6) $$\frac{d\left[Tes\right]}{dt}=K.$$

Solving Equations (5) and (6) gives

(7) [Tes]=KN+C

or

(8) [Tes]=Kt+C.

**Figure 4:**
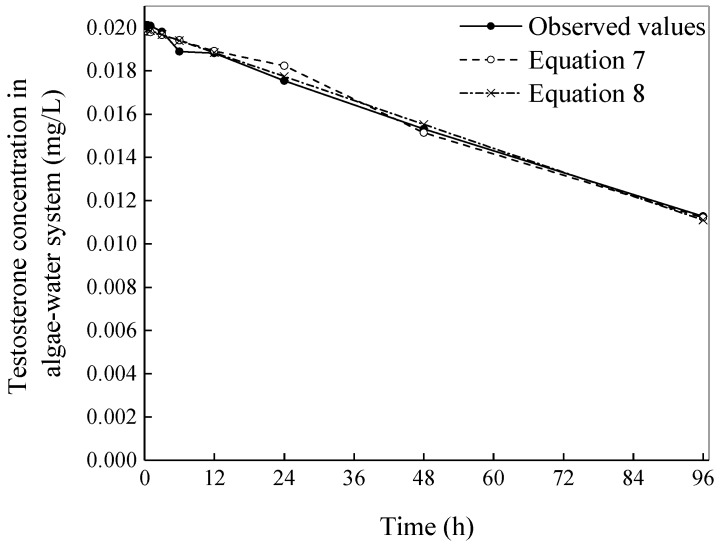
The processes of testosterone biodegradation by *Chlorella vulgaris* with observed values and predicted values fit by Equations (7) and (8).

The relationships between [*Tes*] and *t*, both the observed values and predicted values, are shown in Figure 4. 

The results calculated using Equations (7) and (8), shown in Figure 4, indicate that the relative mean deviations of the testosterone concentrations in the algae–water system of the 0.02 mg/L group between the calculated values and the observed values were 1.55% and 1.18%, *r* = −0.992 and −0.996, respectively. Kinetic Equation (8) could better describe the process of testosterone biodegradation by *C. vulgaris* for the 0.02 mg/L group. Therefore, for a relatively low testosterone concentration, our assumption may be accurate.

The growth equation of algae can be expressed by logistic equations [35] as follows (Equations (9) and (10)):

For the 0.2 mg/L group:

(9) $$N=\frac{56.7577}{1+e^{-0.0493\left(t-45.12811\right)}}.$$

For the 0.02 mg/L group:

(10) $$N=\frac{56.01572}{1+e^{-0.05069\left(t-44.35943\right)}}.$$

After substituting the algae growth equation, changes in the testosterone concentration in the algae–water system with time can be imitated by Equations (11) and (12):

(11) $$0.2\;\mathrm{mg}/L\;\left[Tes\right]=-2.6828\times 10^{-5}{\left(\frac{56.75577}{1+e^{-0.0493\left(t-45.12811\right)}}\right)}^{2}+0.2060$$

(12) $$0.02\;\mathrm{mg}/L\;\left[Tes\right]=-9.2176\times 10^{-5}t+0.01995.$$

## 4. Discussion

The present research is the first to study the accumulation and degradation of testosterone by algae. The results showed that *Chlorella vulgaris* had a significant ability to remove testosterone by bioaccumulation and biodegradation. For both the high-concentration group and the low-concentration group, there was a significant and rapid accumulation at the initial phase of the experiment, followed by a rapid decline of testosterone in algae. The accumulation kinetic of testosterone by *C. vulgaris* is in agreement to the previous report for the accumulation process of several HOCs by phytoplankton [36], which indicated that accumulation occurs in multiple compartments. Considering the testosterone accumulation curves of *C. vulgaris* in Figure 1, the results suggest that there are three stages in the testosterone accumulation process. 

In the first stage, when the density of the algal cells and the rate of algal growth are small, the accumulation is primarily due to surface adsorption. The testosterone diffuses into the algal cell surface quickly while the biodegradation rate is relatively small, which causes the concentration of testosterone accumulated by algae to increase rapidly. In the second stage, the rate of algal growth becomes larger and algal growth has a diluting effect. As a result, the concentration of testosterone accumulated by the algae declines rapidly and reaches its minimum. In the third stage, the rate of algal growth is slower than that in the second stage. With the reduced diluting effects of algal growth, the concentration of testosterone accumulated by the algae increases gradually with time.

It has been suggested that the accumulation of organic compounds in algae is the first step of biomagnification in food webs [37]. This study indicated that a rapid accumulation was found in the early stage of the experiment, and then the concentration of testosterone accumulated by the algae declined rapidly because of the dilute effect of algal growth. However, if the grazing pressure is high in the aquatic ecosystem, there might be significant biomagnification of testosterone along the food chain. Based on the serious threat of environmental androgens to aquatic organisms [5,19,20], a better understanding of trophic transfer of testosterone along food webs will help to assess its ecological risk.

Figure 2 shows that the concentration of total testosterone in the algae–water system significantly decreased with time, indicating that *C. vulgaris* does have a degradation effect on testosterone (*p* < 0.01). Several processes might be involved in the testosterone removal processes, including photodegradation, sorption, and biodegradation [28]. However, little variation in testosterone concentrations in Control I and Control II demonstrates that the phytodegradation plays a small role in the dissipation of testosterone. The results show that the removal of testosterone by algae was mainly caused by biodegradation (69.64% and 42.48%), rather than by accumulation in algal cells. Thus, the testosterone removal mechanisms may involve two processes, a rapid initial adsorption followed by absorption, accumulation and degradation processes.

Many researchers have studied the biodegradation kinetics of organic pollutants by microalgae; however, the biodegradation feature is still a matter of debate. Some researchers reported that the biodegradation by microalgae followed a first-order kinetic model [27,38], whereas some other researchers found that a second-order kinetic equation could describe the biodegradation process better [34].

The degradation results in this study (Figure 2) show a noticeable difference in the degradation between the high-concentration group and the low-concentration group at the end of the experiment, although 0.02 and 0.2 mg/L testosterone had no significant influence on the growth of *C. vulgaris*. Therefore, it is reasonable to suspect that at an equal algae density, different initial concentrations of pollutant could affect the degradation characteristics of the microalgae.

Based on the fitting effects of different dynamic equations on the experiment results, the following hypotheses can be made: the biodegradation rate of testosterone is not only affected by the density of algal cells (*N*) and the growth rate (*r*) of the cells at different growth stages, but it also depends on the relative initial testosterone concentration.

At a high initial testosterone concentration, the biodegradation rate varies with the algal cell density (*N*) and growth rate (*r*). However, at lower testosterone concentrations, the reaction becomes independent of *N* and *r* (zero-order kinetics). In the present study, Equation (11) and Equation (12) could well demonstrate the dynamic process of testosterone biodegradation by *C. vulgaris* with high and low testosterone concentrations, respectively. When the initial testosterone concentration is in the middle range, the reaction (relative to *N* and *r*) may be a mixed-stage reaction. Furthermore, at a certain initial testosterone concentration, as the algal cell density (*N*) and growth rate (*r*) increase, the reaction may gradually change from second-order kinetics to zero-order kinetics. 

The biodegradation of organic contaminants by algae has been demonstrated in several studies. *C. vulgaris* is ubiquitous in water environments and exhibits outstanding potential in the purification of pollutants. Researchers showed that the nonylphenol degradation percentage by *C. vulgaris* was 68.8% [28] and the florfenicol removal efficiency could reach 97% by *Chlorella* sp. L38 [38]. Although the concentrations of testosterone used in the present study are unlikely to be detected in an aquatic ecosystem, *C. vulgaris* demonstrated a high potential capability for testosterone removal, indicating good prospects for use in wastewater treatment. 

With respect to the product of biodegradation, research on the biotransformation of steroid estrogens by *C. vulgaris* indicated that the transformation product exhibited a preference to polar metabolites. Conjugation, which is a rapid detoxification mechanism by increasing the polarity of organic contaminants, plays a major role in biotransformation [31]. To identify the major metabolic products and the biodegradation pathways of testosterone by microalgae, further studies are needed.

## 5. Conclusions

*Chlorella vulgaris* has a significant ability to bioaccumulate testosterone. The experimental data on the amount of testosterone accumulated by algae reveal a sigmoidal pattern, and the degradation of testosterone by *C. vulgaris* was significant. Thus, the algae are useful in testosterone removal. The biodegradation rate of testosterone is not only affected by algal cell density (*N*) and growth rate (*r*) at different growth stages, but it also depends on the relative ratio of the testosterone concentration and algal cell density.

## Figures and Tables

**Table 1:** Experimental design.

Groups	Testosterone Concentration (mg/L)	Initial Density of Algal Cells (cell/mL)
High-concentration	0.2	5 × 10^6^
Low-concentration	0.02	5 × 10^6^
Control I	0.2	〇 ^1^
Control II	0.02	〇
Control III	〇	5 × 10^6^

〇: Null.

**Table 2:** The algal density of *Chlorella vulgaris* under different concentrations of testosterone.

Time (h)	Density of Cells (10^6^ cell/mL)		
	Control III	Low-Concentration Group	High-Concentration Group
0	5.18 ± 0.04	5.21 ± 0.05	5.23 ± 0.02
0.5	5.28 ± 0.02	5.21 ± 0.05	5.30 ± 0.02
1	5.43 ± 0.10	5.21 ± 0.05	5.43 ± 0.02
3	6.02 ± 0.40	6.09 ± 0.00	6.18 ± 0.10
6	7.38 ± 0.17	7.44 ± 0.08	7.46 ± 0.09
12	9.99 ± 0.15	10.18 ± 0.05	10.53 ± 0.10
24	13.71 ± 0.06	13.89 ± 0.04	14.29 ± 0.19
48	30.82 ± 0.15	30.70 ± 0.56	30.40 ± 0.66
96	52.83 ± 0.42	52.18 ± 0.41	52.49 ± 0.32
